# A Network Analysis of Health Care Access and Behavioral/Mental Health in Hispanic Children and Adolescents

**DOI:** 10.3390/bs15060826

**Published:** 2025-06-17

**Authors:** Isis Garcia-Rodriguez, Samuel J. West, Camila Tirado, Cindy Hernandez Castro, Lisa Fuentes, Paul B. Perrin, Oswaldo A. Moreno

**Affiliations:** 1 Department of Psychology, Virginia Commonwealth University, Richmond, VA 23284, USA; garciaroi@vcu.edu (I.G.-R.); tiradoc@vcu.edu (C.T.); hernandezcm3@vcu.edu (C.H.C.); fuentesls@vcu.edu (L.F.); oamoreno@vcu.edu (O.A.M.); 2 Department of Psychology, Virginia State University, Richmond, VA 23806, USA; sawest@vsu.edu; 3 Department of Psychology, University of Virginia, Charlottesville, VA 22904, USA; 4 School of Data Science, University of Virginia, Charlottesville, VA 22903, USA

**Keywords:** barriers, Hispanic youth, NHIS, mental health, psychometric network analysis

## Abstract

Hispanic youth have one of the highest rates of unmet physical and mental health needs. This study aims to examine how child and adolescent healthcare access creates pathways to behavioral/mental health among a national sample of 1711 U.S. Hispanic youth. Using psychometric network analysis, unique pathways in which child healthcare access (i.e., transportation and health service-related factors) and behavioral/mental health were identified. Findings indicate relationships among depression, anxiety, school settings, and friendships. These associations offer a starting point for interventionists and policymakers to ensure that interventions are not targeted individually but from an ecological systems framework. This study may raise awareness of Hispanic youth’s barriers and better equip scientists to plan and implement approaches to address identified barriers.

## 1. Introduction

The Hispanic population^1^ is one of the largest racial/ethnic groups in the United States (U.S.), making up approximately 18.7% of the total population (United States Census Bureau, 2021), and one-third of U.S. Hispanics are under the age of eighteen (Patten, 2016). Despite being one of the largest racial/ethnic populations, Hispanic children and adolescents (HCAs) and their families underutilize healthcare services when compared to other racial/ethnic groups (Bustamante et al., 2019; Langellier et al., 2016; Moreno & Cardemil, 2013; Perez-Escamilla, 2010; Ramírez García, 2019). This underutilization may have increased in recent years since the COVID-19 pandemic (Andraska et al., 2021; Ormiston et al., 2023), anti-immigrant rhetoric (Caballero et al., 2022; Moreno et al., 2024, 2021; Ornelas et al., 2021; Rojas Perez et al., 2023), and xenophobic policies (Blackburn & Sierra, 2021; Slopen et al., 2023) may have invoked uncertainty and distrust in professional settings (Blackburn & Sierra, 2021), and so these unmet child health needs may further exacerbate behavioral/mental health outcomes (Galvan et al., 2024; Slopen et al., 2023). These public health concerns highlight an urgent need to address the physical and mental health needs of HCAs and their caregiving families, especially since social determinant factors are increasing vulnerability to additional physical and mental illnesses (Garcini et al., 2022; Johnson et al., 2023; Mendoza et al., 2024). Although consistent research has documented that HCAs face individual-level (Alegría et al., 2022), community-level (Alegría et al., 2022; Hodgkinson et al., 2017; A. E. West et al., 2023), and systemic-level barriers (Alegría et al., 2022; Hodgkinson et al., 2017; A. E. West et al., 2023), specific paths of HCAs’ healthcare access and behavioral/mental health outcomes remain unexplored. This study will, therefore, conduct a network analysis to examine child health access and behavioral/mental health outcomes specific to HCAs.

### Child Healthcare and Behavioral/Mental Health

Child healthcare, whether physical or mental health-related, is crucial for the development and wellness of every child. Healthcare access for children and adolescents is often defined by whether they have a usual place to receive both preventive and treatment services—an important indicator of access and quality of care (Strickland et al., 2011). However, many healthcare disparities and social determinant structures (e.g., insurance policies) delay or impede these HCAs and their families from receiving adequate medical care. As a result, these healthcare disparities continue to impact HCAs disproportionately. Although there have been numerous strides to reduce this healthcare access, such as the Affordable Care Act (ACA; Alcalá et al., 2017; Ortega et al., 2018), these policies that provide Hispanic families with health insurance and support improved access to child healthcare are being politicized and are at risk of being rescinded (Holahan et al., 2025; Neiman et al., 2021; O’Connor, 2025). These structural barriers, coupled with the individual-level (stigma, class impacting the quality of care, transportation) and community-level barriers (insufficient clinics in Hispanic communities, lack of bilingual providers; Fuentes et al., 2024; Reardon et al., 2017; Santana et al., 2023), all continue to impact child healthcare access and utilization (Alegría et al., 2022; A. E. West et al., 2023). More attention is needed to examine and understand additional factors influencing child healthcare access and utilization in HCAs in these uncertain times.

Examining child healthcare access in HCAs is critical. These youth are at elevated risk for conditions such as depression, anxiety, and behavioral challenges, which are often shaped by their experiences accessing—or being unable to access—adequate healthcare services. Research continues to show that mental health risks are on the rise for HCAs. For example, the 2023 National Survey on Drug Use and Health (SAMHSA, 2024) reported that 20.2% of HCAs experienced a major depressive episode, and 12.4% of HCAs ages 12–17 reported suicide ideations within the last year that the survey was taken, and since recent years anxiety-related symptoms is on the rise (Parodi et al., 2022). Additionally, HCAs reported substance use onset at an earlier age compared to their non-HCAs counterparts (Birdsey et al., 2023; Okine & Unger, 2024). Research also indicates that these pathways to later behavioral/mental health start in early childhood (Anderson & Mayes, 2010). In early childhood, internalizing disorders and externalizing disorders are the most common mental health concern (Gonzales et al., 2017; Isasi et al., 2016; Merikangas et al., 2011), and HCAs are at great risk of these disorders (Bámaca-Colbert et al., 2012).

There are many reasons why early childhood behavioral/mental health outcomes are disproportionately reported in HCAs. Social determinants of health frameworks (E. T. Burton et al., 2024; Tesfaye et al., 2024) suggest that disproportionate stressful life events, also known as adverse childhood experiences (ACEs; Felitti et al., 1998; Wang et al., 2024), are experienced at excessive levels by HCAs. For example, consistent research suggests that neighborhood environments, socioeconomic status, and access to healthcare are commonly encountered among HCAs. Most recently, stressful life events can also include immigration-related factors like fear of deportation, discrimination, and systemic level disadvantages in social and systemic structures/supports (Berge et al., 2020; Walsdorf et al., 2024). These adverse childhood experiences continue to have a positive relationship with behavioral/mental health outcomes in HCAs (Walsdorf et al., 2024). More attention is needed to examine the child healthcare access and behavioral/mental health outcomes in HCAs.

Given the specific paths of child healthcare access and behavioral/mental health outcomes remain unexplored, the purpose of this study is to identify essential patterns of connections among child health access and behavioral/mental health outcomes specific to HCAs. Since Hispanics are the U.S.’s largest racial/ethnic minority group and HCAs have one of the highest rates of unmet mental health needs amongst other marginalized populations (Casseus, 2024; Chang & Slopen, 2024), this study utilizes a network analysis to understand the connections between child healthcare access and behavioral/mental health outcomes. Specifically, this study aimed to identify unique pathways to understand how barriers are associated with depression and anxiety-related symptoms in U.S. HCAs.

## 2. Method

### 2.1. Procedure

This study utilizes data from the 2019 National Health Interview Survey (NHIS), a resource provided by the CDC’s National Center for Health Statistics (NCHS; National Center for Health Statistics, 2020), which documents the health of the non-institutionalized U.S. population. The NHIS, a cross-sectional survey, is designed to capture a snapshot of the health of Americans, utilizes in-home, digital interviews, complemented by telephone interviews when necessary, guaranteeing accessibility and completeness across the 50 states and the District of Columbia (excluding Puerto Ricans living in Puerto Rico). For more thorough information about the NHIS sampling and data collection procedure, please visit https://www.cdc.gov/nchs/nhis/documentation/2019-nhis.html?CDC_AAref_Val=https://www.cdc.gov/nchs/nhis/2019nhis.htm (accessed on 22 August 2024).

### 2.2. Participants

The publicly released data files for the 2019 NHIS contained data for 33,138 households containing 31,997 adults (National Center for Health Statistics, 2020). The number of youth in the sample was 9193, of whom 2173 were identified within the Hispanic subgroup (23.64% of the total “sample child” sample). Of this sub-sample (*n* = 2173), the current study retained a final sample of 1711 youth aged 4–17, removing any participants younger than four based on the age requirement for the mental health variables.

The sample ranged in age from 4 to 17 years; the mean age was 10.9 years (*SD* = 4.05), and the majority (53%) were males. In total, 92.6% of the sample were U.S.-born, and 31.3% of the family’s yearly income was reported to be between USD 0 and USD 34,000. In total, 48.5% of the sample reported having Medicaid or some other public health insurance option, with 40.3% having private insurance and 8.2% being uninsured. Approximately 1% of the sample reported they both delayed mental health treatment for their child, or the child did not receive mental health treatment due to costs. Also, 19% of the sample reported being very worried about paying medical bills, 24.5% were somewhat worried, and 56% were not worried. It should also be noted that 42.7% of the sample resided in large, central metropolitan cities. Descriptive statistics of all variables can be found in Table 1.

### 2.3. Measures

The NHIS (before 2019) consists of four survey modules including household composition, family core, sample adult, and sample child. The sample child section of the 2019 NHIS covers additional subject areas not included in the family core, which collects information on all members of a household. The questions in the sample child section are more specific and are intended to gather more detailed information than those in the family core. The sections include child identification section (CID), health status and conditions (HSCs), functioning and disability (FD), health care access and health service utilization (CAU), behavioral and mental health (BMH), and stressful life events (SLEs). The current study used only select data from the CID, CAU, BMH, and SLE sections.

#### 2.3.1. Child Identification Section (CID)

The CID contains information about the availability of a respondent knowledgeable about the sampled child’s health, including that person’s relationship to the sample child. This section also includes a variable indicating whether the sample child questionnaire started two or more weeks after the initial interview. The CID section also contains questions that verify the sample child’s sex, age, and date of birth, which were initially provided by the family respondent earlier in the interview.

#### 2.3.2. Child Health Care Access and Utilization Section (CAU)

The CAU contains information on access to healthcare and healthcare provider contacts. The questions on access to health care include having a usual place for sick care (place_sick), having a typical place for routine/preventive care (place_routine), change in place of care (place_change), reasons for a delay in receiving medical care (delay_care), and the inability to afford medical care (afford_care). Variables pulled from the CAU section were considered access and utilization barriers.

#### 2.3.3. Behavioral and Mental Health (BMH)

The purpose of the BMH is to provide information on youth’s behavior as measured by the Strengths and Difficulties Questionnaire (SDQ) (Goodman, 1997, 1999). The SDQ is a behavioral screening questionnaire for youth aged 4 to 17 years with extended questions that provide information on the duration of a child’s problem and the impact that the problem has on the child and his/her family. All 25 questions of the SDQ are included in the 2019 NHIS, along with five additional measures of emotionality, conduct, hyperactivity, peer problems, and prosocial behavior. Variables pulled from the BMH section were operationalized as mental health outcomes.

#### 2.3.4. Stressful Life Events (SLEs)

The SLE includes four questions to understand if a child has experienced specific stressful life events, also known as adverse childhood experiences (ACEs). The questions assess if the child has experienced neighborhood violence and if they have lived with someone who has been incarcerated, mentally ill, and/or someone with a drug or alcohol problem. Responses to the four SLE questions were also considered mental health outcomes.

## 3. Analysis

To model connections between unmet mental health needs and mental health, network analysis was used. Networks can be best estimated on cross-sectional or longitudinal data at the group or individual level (Epskamp et al., 2018; Hevey, 2018; Rhemtulla et al., 2016). Networks can be used as tools to address multicollinearity and predictive mediation, as well as highlighting the presence of latent variables (Epskamp et al., 2018). Network analysis is a hypothesis-generating methodology used in studies similar to the current research (Allen et al., 2008; Goodall et al., 2014; Kanamori et al., 2019). The analysis uses partial correlation networks paired with machine learning applied during regularization techniques to reveal patterns of unique associations often obscured by various factors in more traditional analyses. This allows researchers to represent individual items in the context of a dynamic system to visually examine the covariance structures among individual indicators (often items used to measure an ostensible latent construct). This is beneficial because it is a theoretically neutral approach to exploring such data and has facilitated critical advances in the domains of rehabilitation medicine, psychopathology, and personality (e.g., Klyce et al., 2021; Robinaugh et al., 2020; S. J. West & Chester, 2021).

Variables in such networks are reflected visually as “nodes,” whereas the partial associations between any pair of nodes are reflected by the “edges” or the paths that connect the nodes. To estimate the network, a Graphical Gaussian Model (GGM) was used (Lauritzen, 1996) in which edges represent conditional independence relationships among the nodes. These edges can be understood as partial correlations, representing the relationship between two nodes when controlling for all other relationships in the network. GGMs estimate many parameters that likely result in some false positive edges. Therefore, it is common to regularize GGMs via the Graphical Least Absolute Shrinkage and Selection Operator algorithm (*glasso*; Friedman et al., 2010; Tibshirani, 1996). This algorithm shrinks all edges in the network. It sets small edges to zero, which leads to a more parsimonious network that explains the covariance among nodes with as few spurious edges as possible.

The GGM was estimated using the R-package *bootnet* (Epskamp et al., 2012) that uses the Extended Bayesian Information Criterion (*EBIC*) model selection function by default to implement the *glasso* regularization. For network visualization, the edges’ thickness represents the association’s magnitude, and the edges’ color represents the direction of the relationship (i.e., red = negative, blue = positive). The R (R Core Team, 2021) package *qgraph* (Epskamp et al., 2012) was used to calculate and visualize the networks.

### 3.1. Centrality

Several indices of node centrality were calculated to identify which variables are most central to the network (Opsahl et al., 2010). Strength centrality indexes the overall influence of a single node in a given network. Computationally, strength centrality is estimated as the sum of all edges connected to a given node. We have focused exclusively on strength centrality as other centrality indices are challenging to interpret in cross-sectional networks (Hallquist et al., 2021).

### 3.2. Stability

To indicate whether the order of centrality indices remains the same after re-estimating the network with fewer participants, a case-dropping bootstrap was used to check for stability (*bootnet* package). The procedure estimated 5000 sample datasets to estimate the network, repeatedly dropping participants. The minimum value for stability was set at 0.25, with 0.75 being the highest possible value (Epskamp et al., 2018).

## 4. Results

### 4.1. Hispanic Child Network

**Figure 1:**
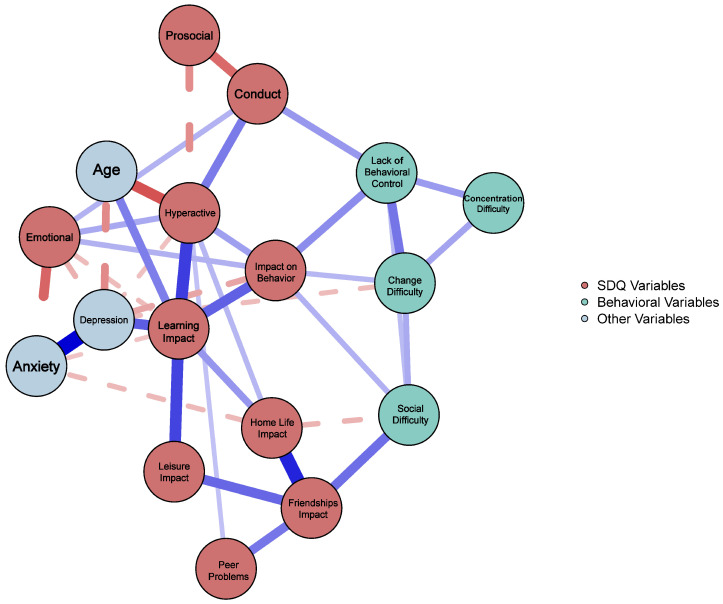
Psychometric network. *Note.* Figure 1 depicts a psychometric network analysis of the 17 variables. Each variable is depicted using a circle, where red circles represent SDQ variables, green circles represent behavioral variables, and blue circles represent other variables. Positive associations are represented by the blue lines connecting circles, and negative associations are represented by red lines. The thickness of each line is representative of the strength of the pathways.

Figure 1 shows a visualization of the network structure of the child identification (CID), child access and utilization (CAU), behavioral and mental health (BMH), and stressful life events (SLEs) variables that were used. Overall, variables were positively connected within the network. Powerful connections emerged between difficulties interfering with home life (HomeLife) and difficulties interfering with friendships (Friendships), anxiety (Anx.) and depression (Dep.), difficulties interfering with classroom learning (Classroom) and depression, and a strong negative connection emerged between age (Age) and hyperactivity (Hyper.).

The five nodes with the highest node strength centrality were difficulties interfering with classroom learning (Classroom), hyperactivity (Hyper), depression (Dep.), difficulties with emotions and behavior (Diff_EmoBeh.), and emotionality (Emotionality). In contrast, the two least central nodes were difficulty concentrating (Concentration) and prosocial behaviors (Prosocial). Node centrality estimates for all nodes can be found in Table 2. For a breakdown of the zero-order correlations amongst all study variables, please see Table S1.

### 4.2. Network Accuracy and Stability

The accuracy and stability of the estimated networks were then calculated. The edge weight bootstrap revealed that the network is moderately accurately estimated. For a complete description of the full set of bootstrapped edge weights, along with the edge weights estimated in the network, please see Table S2. The subset bootstrap showed that the order of node closeness centrality is more stable than the order of strength and betweenness. This is consistent with the CS coefficient, which was 0.52 for node strength.

## 5. Discussion

This study represents a network analysis of HCAs and is one of the first studies to connect their barriers to mental health care and outcomes. The primary aim of this study was to identify unique pathways and barriers to mental health care for HCAs. Furthermore, this study is intended to guide future researchers and interventionists toward developing ways to address such obstacles on micro and macro levels so Hispanic youth can access and utilize services to meet their needs. This aim is unique to the network analysis literature and, therefore, adds to the small body of literature on healthcare access networks, specifically within the Hispanic population residing in the U.S.

### 5.1. Hispanic Child Network

In the current study, variables were generally positively connected. The most substantial edges in the network emerged between difficulties interfering with home life (HomeLife) and difficulties interfering with friendships (Friendships); anxiety (Anx.) and depression (Dep.); difficulties interfering with classroom learning (Classroom) and depression; and a strong negative connection emerged between age (Age) and hyperactivity (Hyper.). However, some variables provided weak connections with others, such as prosocial behavior and emotionality.

Anxiety (Anx.) and depression (Dep.) were highly connected in the network, as well as age (Age) and hyperactivity (Hyper.). The connections between these variables have important implications for working with this community. For example, racial/ethnic minority youth, including HCAs, are commonly labeled as disruptive in classroom settings (Alegría et al., 2010; Leath et al., 2019). Racial/ethnic minority youth also often experience “adultification” bias, where youth are treated as more mature than they are by a reasonable standard of development (L. Burton, 2007; Haerle, 2019; Kuperminc et al., 2013). The correlation between age and hyperactivity was negative, such that as age increases, hyperactivity decreases. It is critical that school personnel understand this developmental trend, as racial/ethnic minority youth—particularly Hispanic children and adolescents—are often mischaracterized as disruptive or aggressive, leading to disproportionate disciplinary actions or referrals to juvenile justice systems (e.g., Haerle, 2019).

Many Hispanic families face barriers to care rooted in cultural values, especially stigma (Gearing et al., 2024). Turner et al. (2015) demonstrated that stigma is significantly associated with a reduced likelihood of help-seeking as reported by Hispanic American parents, underscoring the need to address culturally rooted concerns in mental health outreach and intervention. Moreover, the observed link between depression (Dep.) and difficulties interfering with classroom learning (Classroom) presents an important opportunity for early identification and intervention. This association is particularly relevant for caregivers and educators, who may be in key positions to notice academic struggles that reflect underlying psychological distress. Prior research has also noted that Hispanic individuals delay help-seeking until they experience physiological symptoms (e.g., fatigue, heart palpitations, headache) of depression and anxiety (Dunlop et al., 2020; Grassie et al., 2022). For HCAs, academic difficulties may serve as early indicators of mental health concerns. Therefore, integrating mental health screenings into educational settings, along with providing culturally informed psychoeducation to families, may help bridge the gap between need and service use.

The connection between difficulties interfering with home life (HomeLife) and difficulties interfering with friendships (Friendships) was not as intuitive as the previously described connections, though it has been seen in previous literature (Criss et al., 2002; Schwartz et al., 2000). A study by Criss et al. (2002) found that peer acceptance and friendship were protective factors of family adversity and child externalizing behaviors. The connection found in the current study is relevant in that more attention should be paid to the relationships that youth hold with classmates, especially within Hispanic child and adolescent populations. Within the Hispanic community, familism can be a protective and risk factor, depending on family dynamics (Stein et al., 2015; Valdivieso-Mora et al., 2016). Therefore, if youth hold positive, mutually empathetic relationships with peers, this can serve as a protective factor against family adversity. This association also may prove crucial for youth in mixed-status families in the face of immigration-related stressors.

### 5.2. Limitations, Strengths, and Future Directions

The network included in this study included dichotomous data, which are a better fit with an Ising Model (Cipra, 1987) or Mixed Graphical Model (MGM; Lee & Hastie, 2015), rather than a GGM which utilizes partial Spearman’s rank-order correlations to assess for associations. Future research may consider running a similar network with a different model. The sample size was a strength in that it was large enough for inadequate psychometric network analysis, and it generated enough power to move forward with the analysis. Given the cross-sectional nature of the NHIS data used in the current study, our findings do not allow for inferences about causal or temporal relationships among variables. Future studies utilizing longitudinal designs, whether through panel datasets or linked NHIS waves, are critically needed to better understand how child healthcare access and behavioral/mental health symptoms evolve over time within Hispanic populations.

Future research may also consider continuing to assess the impact of peer relationships on Hispanic children and adolescents, particularly as it relates to cultural values such as familism, stigma, and gender roles. Clinical future directions include assessing differences between demographically different groups of youth (e.g., racial/ethnic minority groups, White groups, etc.) to add to existing interventions and create new ones. By identifying barriers to mental health care across racial/ethnic groups, interventions can become more targeted. Due to the high centrality of the variables related to school difficulties, interventions should target school systems to increase the potential for efficacious treatments. For clinicians, identifying barriers to access and utilization allows for creative and alternative methods of meeting clients where they are.

### 5.3. Conclusions

The HCA population is growing exponentially daily, and alongside it, the rates of unmet behavioral/mental health needs. Using a nationally representative sample of HCAs, these findings suggest that barriers to care can be addressed through school systems and social networks. Ensuring HCAs meet their basic needs includes offering solutions at accessible levels for all youth, such that initiatives that center equity will enhance the lives of many.

## Figures and Tables

**Table 1:** Variable descriptives.

*Variable*	*M*	*SD*	*Min*	*Max*	*Skew*	*Kurtosis*
Age	10.99	4.05	4	17	−0.19	−1.19
Anxiety	4.33	1.12	1	5	−1.76	2.09
Change Difficulty	1.21	0.51	1	4	2.63	7.19
Concentration Difficulty	1.10	0.39	1	4	4.43	21.87
Conduct	0.86	1.32	0	9	2.03	5.00
Depression	4.64	0.83	1	5	−2.69	7.07
Emotional	1.22	1.72	0	10	1.87	3.81
Friendships Impact	1.56	0.83	1	4	1.41	1.16
Home Life Impact	1.73	0.86	1	4	0.98	0.13
Hyperactive	2.05	2.32	0	10	1.33	1.36
Impact on Behavior	1.29	0.59	1	4	2.21	5.08
Lack Behavioral Control	1.18	0.50	1	4	3.29	11.89
Learning Impact	2.07	1.08	1	4	0.56	−1.03
Leisure Impact	1.46	0.79	1	4	1.73	2.20
Peer Problems	1.26	1.54	0	10	1.60	3.24
Prosocial	8.91	1.72	0	10	−2.11	5.18
Social Difficulty	1.12	0.41	1	4	4.03	18.26

**Table 2:** Node centrality estimates.

Node	Betweenness	Closeness	Strength	Expected Influence
Prosocial	0	0.005	0.41	−0.41
Peer	2	0.005	0.58	0.58
Hyper	16	0.007	1.51	0.58
Conduct	14	0.006	0.83	0.45
Emotionality	4	0.005	0.97	0.11
Age	0	0.006	0.62	−0.24
Leisure	10	0.006	0.61	0.39
Classroom	24	0.007	1.58	1.18
Friendships	14	0.006	0.97	0.97
HomeLife	2	0.005	0.81	0.37
Diff_EmoBeh	13	0.006	1.02	0.75
MakeFriends	6	0.005	0.67	0.45
ChangeRoutine	5	0.005	0.69	0.48
Concentration	0	0.004	0.29	0.29
Control	12	0.005	0.78	0.78
Depression	10	0.006	1.23	0.02
Anxiety	4	0.005	0.80	−0.03

## Data Availability

Data used in this study are publicly available from the following website: https://www.cdc.gov/nchs/nhis/documentation/index.html (accessed on 22 August 2024).

## Note

1 Hispanic and Latina/o are used interchangeably to refer to Latin Americans and/or individuals who speak Spanish. In efforts to stay consistent with the term from the parent study, we kept the term Hispanic instead of using Latina/o or Latinx to describe the population. However, when using Hispanic, we recognize and honor the gender-expansive (i.e., Latinx) and Afro-Latin individuals who are also a part of the Hispanic/Latinx communities. See Cardemil et al. (2019) for more information.

## Supplementary Materials

The following supporting information can be downloaded at: https://www.mdpi.com/article/10.3390/bs15060826/s1, Table S1: Zero-order Spearman’s Rank-Order Correlations; Table S2: Edge Weight Estimates and Bootstrap Results for All Edges Included in the Network.
