# Supplementary material for: A Network Analysis of Health Care Access and Behavioral/Mental Health in Hispanic Children and Adolescents
Source: Behav Sci (Basel). 2025 Jun 17;15(6):826. doi: 10.3390/bs15060826 (PMC12189966; doi:10.3390/bs15060826)
Supplement: Supplementary file 1 [file behavsci-15-00826-s001.zip › behavsci-3554152-supplementary.pdf]

Supplemental Table S1. Zero-order Spearman's Rank-Order Correlations.

		1	2	3	4	5	6	7	8	9	10	11	12	13	14	15	16
1	Age																
2	Anxiety	-0.15***															
3	Change Difficulty	-0.01	-0.31***														
4	Concentration Difficulty	-0.05	-0.21***	0.35***													
5	Conduct	-0.10***	-0.22***	0.32***	0.20***												
6	Depression	-0.18***	0.54***	-0.31***	-0.12***	-0.21***											
7	Emotional	0.03	-0.47***	0.35***	0.21***	0.35***	-0.41***										
8	Friendships Impact	0.05	-0.30***	0.30***	0.25***	0.22***	-0.19***	0.30***									
9	Home Life Impact	0.11*	-0.34***	0.28***	0.19***	0.25***	-0.22***	0.30***	0.48***								
10	Hyperactive	-0.17***	-0.28***	0.40***	0.30***	0.49***	-0.25***	0.39***	0.26***	0.36***							
11	Impact on Behavior	0.01	-0.38***	0.46***	0.32***	0.39***	-0.35***	0.42***	0.35***	0.34***	0.52***						
12	Lack Behavioral Control	-0.06*	-0.22***	0.46***	0.35***	0.39***	-0.19***	0.25***	0.28***	0.29***	0.40***	0.47***					
13	Learning Impact	0.10*	-0.25***	0.31***	0.26***	0.26***	-0.04	0.19***	0.36***	0.42***	0.48***	0.48***	0.32***				
14	Leisure Impact	0.02	-0.22***	0.28***	0.18***	0.22***	-0.14**	0.25***	0.45***	0.36***	0.30***	0.33***	0.28***	0.47***			
15	Peer Problems	0.09***	-0.24***	0.23***	0.12***	0.29***	-0.25***	0.31***	0.36***	0.17***	0.30***	0.27***	0.21***	0.20***	0.27***		
16	Prosocial	0.02	0.08***	-0.18***	-0.13***	-0.37***	0.08***	-0.14***	-0.15**	-0.15**	-0.35***	-0.23***	-0.23***	-0.17***	-0.22***	-0.23***	
17	Social Difficulty	0.05*	-0.25***	0.36***	0.26***	0.21***	-0.25***	0.27***	0.38***	0.16**	0.25***	0.38***	0.33***	0.23***	0.22***	0.29***	-0.17***

Note. *$p < .05$, **$p < .01$, ***$p < .001$.

Supplemental Table S2. Edge Weight Estimates and Bootstrap Results for All Edges Included in the Network

Edge	Weight	M	SD	LCI	UCI	P0
Change Difficulty--Concentration Difficulty	0.14	0.12	0.06	0.01	0.26	0.20
Change Difficulty--Depression	-0.10	-0.07	0.06	-0.22	0.02	0.39
Change Difficulty--Lack Behavioral Control	0.20	0.20	0.04	0.13	0.28	0.00
Concentration Difficulty--Lack Behavioral Control	0.15	0.14	0.06	0.03	0.27	0.12
Conduct--Emotional	0.11	0.10	0.05	0.01	0.22	0.21
Conduct--Lack Behavioral Control	0.15	0.15	0.03	0.09	0.22	0.02
Depression--Age	-0.17	-0.17	0.04	-0.24	-0.10	0.01
Depression--Anxiety	0.38	0.38	0.03	0.32	0.44	0.00
Emotional--Anxiety	-0.23	-0.23	0.03	-0.29	-0.17	0.00
Emotional--Depression	-0.12	-0.10	0.05	-0.22	-0.01	0.18
Emotional--Impact on Behavior	0.11	0.10	0.06	0.00	0.23	0.21
Emotional--Learning Impact	-0.10	-0.08	0.08	-0.26	0.06	0.46
Friendships Impact--Home Life Impact	0.33	0.32	0.05	0.22	0.43	0.00
Friendships Impact--Social Difficulty	0.21	0.21	0.06	0.09	0.34	0.02
Home Life Impact--Anxiety	-0.11	-0.09	0.07	-0.25	0.04	0.37
Home Life Impact--Social Difficulty	-0.11	-0.08	0.08	-0.27	0.05	0.44
Hyperactive--Age	-0.26	-0.25	0.03	-0.32	-0.19	0.00
Hyperactive--Conduct	0.20	0.20	0.03	0.13	0.26	0.00
Hyperactive--Depression	-0.09	-0.08	0.06	-0.21	0.02	0.33
Hyperactive--Emotional	0.13	0.12	0.04	0.04	0.21	0.07
Hyperactive--Home Life Impact	0.11	0.09	0.07	-0.03	0.25	0.32
Hyperactive--Impact on Behavior	0.14	0.13	0.05	0.04	0.24	0.06
Hyperactive--Learning Impact	0.29	0.29	0.06	0.18	0.40	0.00
Impact on Behavior--Change Difficulty	0.11	0.09	0.06	-0.01	0.23	0.25
Impact on Behavior--Depression	-0.13	-0.13	0.05	-0.24	-0.03	0.09
Impact on Behavior--Lack Behavioral Control	0.17	0.17	0.04	0.09	0.25	0.02
Impact on Behavior--Social Difficulty	0.12	0.10	0.06	-0.01	0.25	0.28
Learning Impact--Age	0.19	0.19	0.07	0.06	0.32	0.05
Learning Impact--Anxiety	-0.09	-0.07	0.07	-0.23	0.05	0.44
Learning Impact--Depression	0.24	0.24	0.06	0.12	0.36	0.01
Learning Impact--Home Life Impact	0.16	0.14	0.06	0.03	0.28	0.10
Learning Impact--Impact on Behavior	0.24	0.24	0.07	0.10	0.37	0.01
Leisure Impact--Friendships Impact	0.23	0.22	0.06	0.10	0.35	0.01
Leisure Impact--Learning Impact	0.28	0.27	0.05	0.18	0.38	0.00
Peer Problems--Friendships Impact	0.20	0.20	0.06	0.09	0.32	0.02
Peer Problems--Hyperactive	0.09	0.06	0.06	-0.03	0.21	0.45
Prosocial--Conduct	-0.22	-0.22	0.03	-0.27	-0.17	0.00

Prosocial--Hyperactive	-0.17	-0.17	0.03	-0.24	-0.10	0.01
Social Difficulty--Change Difficulty	0.13	0.11	0.06	0.00	0.25	0.24
Social Difficulty--Lack Behavioral Control	0.10	0.07	0.07	-0.03	0.23	0.49

Notes. LCI = Lower-bound of the confidence interval, UCI = Upper-bound of the confidence interval, P0 = proportion of the 5,000 sample bootstrap that did not contain a given edge.
